# PGA_1_-induced apoptosis involves specific activation of H-Ras and N-Ras in cellular endomembranes

**DOI:** 10.1038/cddis.2016.219

**Published:** 2016-07-28

**Authors:** B Anta, A Pérez-Rodríguez, J Castro, C A García- Domínguez, S Ibiza, N Martínez, L M Durá, S Hernández, T Gragera, D Peña-Jiménez, M Yunta, N Zarich, P Crespo, J M Serrador, E Santos, A Muñoz, J L Oliva, J M Rojas-Cabañeros

**Affiliations:** 1 Unidad de Biología Celular, Unidad Funcional de Investigación de Enfermedades Crónicas (UFIEC), Instituto de Salud Carlos III, Madrid 28220, Spain; 2 Fundación Centro Nacional de Investigaciones Cardiovasculares (CNIC), Madrid 28029, Spain; 3 Departamento de Biologia Molecular, Facultad de Medicina, Instituto de Biomedicina y Biotecnología de Cantabria, Consejo Superior de Investigaciones Cientificas (CSIC)-IDICAN, Universidad de Cantabria, Santander 39011, Spain; 4 Departamento de Biología Celular e Inmunología, Centro de Biología Molecular Severo Ochoa (CBMSO), CSIC-UAM, Madrid 28049, Spain; 5 Centro de Investigación del Cancer, IBMCC (CSIC-USAL), Universidad de Salamanca, Salamanca 37007, Spain; 6 Instituto de Investigaciones Biomédicas ‘Alberto Sols', Consejo Superior de Investigaciones Científicas, Universidad Autónoma de Madrid, Arturo Duperier 4, Madrid 28029, Spain

## Abstract

The cyclopentenone prostaglandin A_1_ (PGA_1_) is an inducer of cell death in cancer cells. However, the mechanism that initiates this cytotoxic response remains elusive. Here we report that PGA_1_ triggers apoptosis by a process that entails the specific activation of H- and N-Ras isoforms, leading to caspase activation. Cells without H- and N-Ras did not undergo apoptosis upon PGA_1_ treatment; in these cells, the cellular demise was rescued by overexpression of either H-Ras or N-Ras. Consistently, the mutant H-Ras-C118S, defective for binding PGA_1_, did not produce cell death. Molecular analysis revealed a key role for the RAF-MEK-ERK signaling pathway in the apoptotic process through the induction of calpain activity and caspase-12 cleavage. We propose that PGA_1_ evokes a specific physiological cell death program, through H- and N-Ras, but not K-Ras, activation at endomembranes. Our results highlight a novel mechanism that may be of potential interest for tumor treatment.

The mammalian genome contains three *ras* genes that encode the 21-kDa proteins H-Ras, N-Ras, and K-Ras with their two isoforms, K-Ras4A and K-Ras4B, which are generated from two alternative fourth exons. Ras proteins are small GTPases that act as molecular switches connecting a wide spectrum of extracellular signals from cell-surface receptors to intracellular pathways to control cell proliferation, differentiation, senescence, and death.^1^ Ras proteins are activated by guanine nucleotide-exchange factors, which promote the exchange of guanosine diphosphate (GDP) for guanosine triphosphate (GTP), resulting in a conformational change of the tertiary structure of Ras and exposing its effector loop to interacting partners. The intrinsic GTPase activity of Ras proteins stimulated by GTPase-activating proteins restores the GDP-bound state and terminates Ras signaling. Active GTP-bound Ras interacts with effector proteins that modulate different signaling pathways to generate specific biological outcomes. As Ras proteins are ubiquitously expressed (except K-Ras4A)^2^ and share a high degree of sequence homology and a large number of molecular activators, it was long assumed that their role was redundant. However, functional redundancy is not complete, as demonstrated by embryonic lethality in K-Ras knockouts^3, 4^ and the demonstration that the three Ras proteins have specific roles according to Ras isoform-dependent subcellular compartmentalization.^5^ This observation implies that biochemical and biophysical aspects of specific subcellular sites determine both the Ras isoform and the set of effectors that could be recruited, thus generating different molecular and biological outputs.^6^ Cyclopentenone prostaglandins (CyPGs) are eicosanoids with a varied spectrum of biological activity, including anti-inflammatory and antitumor effects, induction of oxidative stress, modulation of heat-shock response (HSP), and anti-viral activity.^7, 8^ They are thought to originate from the free radical-induced peroxidation of arachidonic acid (isoprostane pathway)^9^ and the dehydration of prostaglandins.^10^ CyPG contain an α,*β*-unsaturated carbonyl group in the cyclopentane ring that favors the formation of Michael adducts with sulfhydryl groups of proteins.^11^ This mechanism is responsible for many of the biological properties of these compounds. However, the variety of cellular responses to CyPG appears to be cell type-specific and concentration-dependent; indeed, cytoprotective and cytotoxic responses have been associated with low and high concentrations of CyPG, respectively.^12, 13^ In this regard, we reported that H-Ras is a target for the addition of a typical CyPG, the 15-deoxy-12,14-prostaglandin J_2_ (15d-PGJ_2_), and that this effect is associated with specific activation of H-Ras-dependent pathways and increased proliferation and inhibition of apoptosis in NIH3T3 fibroblasts^14^ and MCA3D keratinocytes.^15^ Nevertheless, members of the prostaglandin A and J series act as potent inhibitors of some human tumors both *in vitro* and *in vivo*, by inducing cell cycle arrest and apoptosis.^7, 10^ In endothelial cells, activation of peroxisome proliferator-activated receptor (PPAR) receptors by 15d-PGJ_2_ induces nuclear localization of receptors and caspase-mediated apoptosis,^16^ and both 15d-PGJ_2_ and cyclopentenone prostaglandin A1 (PGA_1_) induce apoptosis in AGS cells by a PPAR-independent mechanism.^17^ Although the role of CyPG as inducers of apoptosis is well documented, the relationship between Ras activation by CyPG and triggering of apoptosis, or proliferation, is not fully understood. We have explored the molecular mechanisms underlying PGA_1_-induced apoptosis. We found that PGA_1_ induces apoptosis in mouse fibroblasts by specific binding to and activation of H- and N-Ras at endomembranes, and that this process requires activation of extracellular signal-regulated kinases (ERKs). A detailed analysis revealed that the mechanism of cell death induced by PGA_1_ requires ERK-mediated activation of calpain, an endoplasmic reticulum (ER) protease, and leads to a caspase-dependent apoptosis. Finally, we present evidence that the mechanism of apoptosis is shared by human cancer cells.

## Results

### PGA_1_ induces apoptosis in MEFs

**Figure 1:**
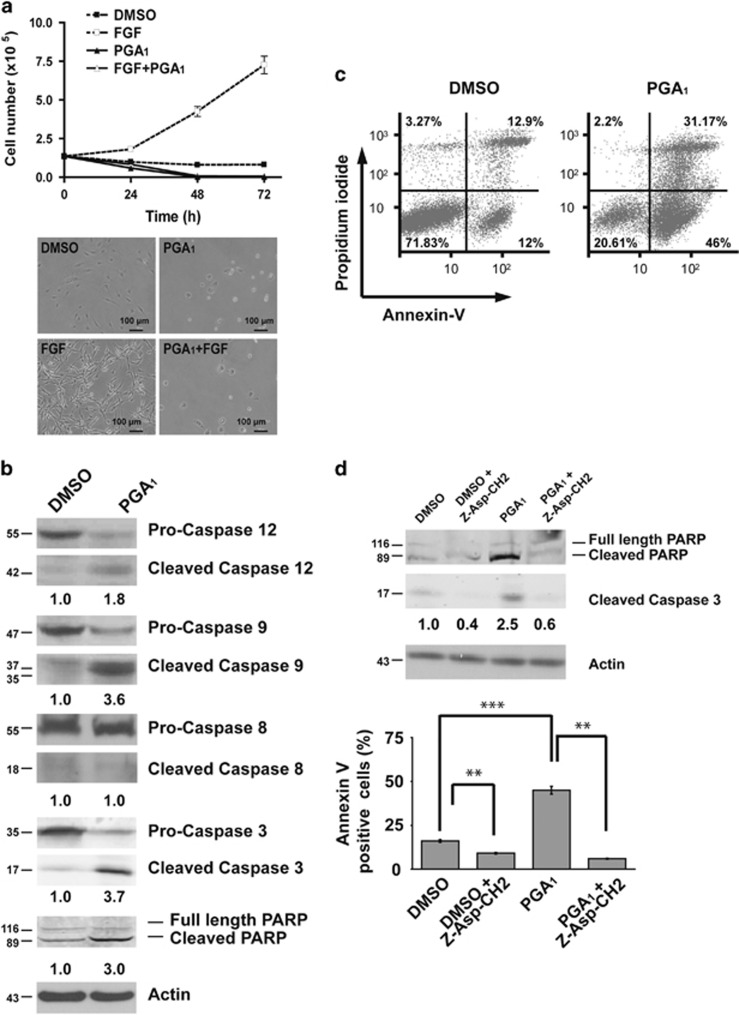
PGA_1_ promotes apoptosis in mouse fibroblasts. (**a**) Serum-starved NIH3T3 cells were treated with 30 *μ*M PGA_1_ (Supplementary Figure S1A), 50 ng/ml FGF, or DMSO every 24 h. (Upper panel) Cells were collected at 24, 48, or 72 h and scored by cell counting; data are presented as mean±S.D. (*n*=3). (Lower panel) Cell morphology was recorded by phase-contrast micrograph 24 h after treatment. (**b**) Serum-starved cultures of wt MEFs were stimulated with 30 *μ*M PGA_1_ or DMSO and harvested 3 h after treatment. Lysates prepared from cells were analyzed by western blotting for procleaved and cleaved forms of caspases and PARP. *β*-Actin (actin) was used as a loading control, and the levels of cleaved caspase-3, which were determined by densitometry, are provided at the bottom (S.D. <10% average in each case). (**c**) At 6 h after treatment with 30 *μ*M PGA_1_ or DMSO, wt MEFs were analyzed for apoptosis using annexin V/FITC staining in a FACS flow cytometer and the percentage of apoptosis was determined. (**d**) Wt MEFs stimulated with 30 *μ*M PGA_1_ or DMSO in the presence or absence of 20 *μ*M Z-Asp-CH2-DCB were harvested 3 h after treatment. (Upper panel) Cell lysates were prepared and analyzed as in (**b**). The levels of cleaved caspase-3, determined by densitometry, are provided at the bottom (S.D. <10% average in each case). (Lower panel) Annexin V/FITC staining analysis was performed as in (**c**) and quantitative analysis of the percentage of the early apoptotic cells. Results are expressed as mean±S.D. (*n*=4), ***P≤0.001 and **P≤0.01. All experiments (**c** and **d**) were carried out four times with similar results

The CyPGs display potent antiproliferative activity in various cellular models. These compounds induce cell cycle arrest or apoptosis depending on the cell type and treatment conditions.^18, 19^ Synchronously starved NIH3T3 cells were incubated in the presence of PGA_1_ to assess antiproliferative activity (according to the scheme shown in Supplementary Figure S1A). Cell number decreased sharply: no viable cells remained after 48 h of treatment, even in the presence of strong mitogenic signals such as fibroblast growth factor (FGF) (Figure 1a). Flow cytometry analysis of propidium iodide-stained cells revealed that ~75% of PGA_1_-treated cells showed an increase in cells with hypodiploid DNA content after 24 h (Supplementary Figure S1B). In addition, DAPI (4′, 6-diamidino-2-phenylindole) staining also revealed apoptotic nuclear morphology after 24 h (Supplementary Figure S1C). Caspase cleavage was examined to verify that the effects of PGA_1_ reflected apoptosis. Western blotting using antibodies that detect both procaspase and activated cleaved forms revealed the presence of cleaved caspase-12, -9, and -3 in cell lysates from PGA_1_-treated cells, as well as cleaved poly(ADP-ribose) polymerase (PARP), a typical target for activated caspase-3 (Figure 1b). Moreover, both annexin V-binding analysis (Figure 1c) and DNA laddering (Supplementary Figure S1D) indicated that this cell death was due to an apoptotic process. In agreement with these data, both caspase-3 activation and the increase in annexin V-positive cells were completely abolished by the pancaspase inhibitor Z-Asp-CH2-DCB (Figure 1d). Taken together, these results demonstrate that treatment of mouse fibroblast cells with PGA_1_ leads to apoptosis through activation of a caspase-dependent pathway.

### PGA_1_-induced apoptosis requires expression of H- and N-Ras

**Figure 2:**
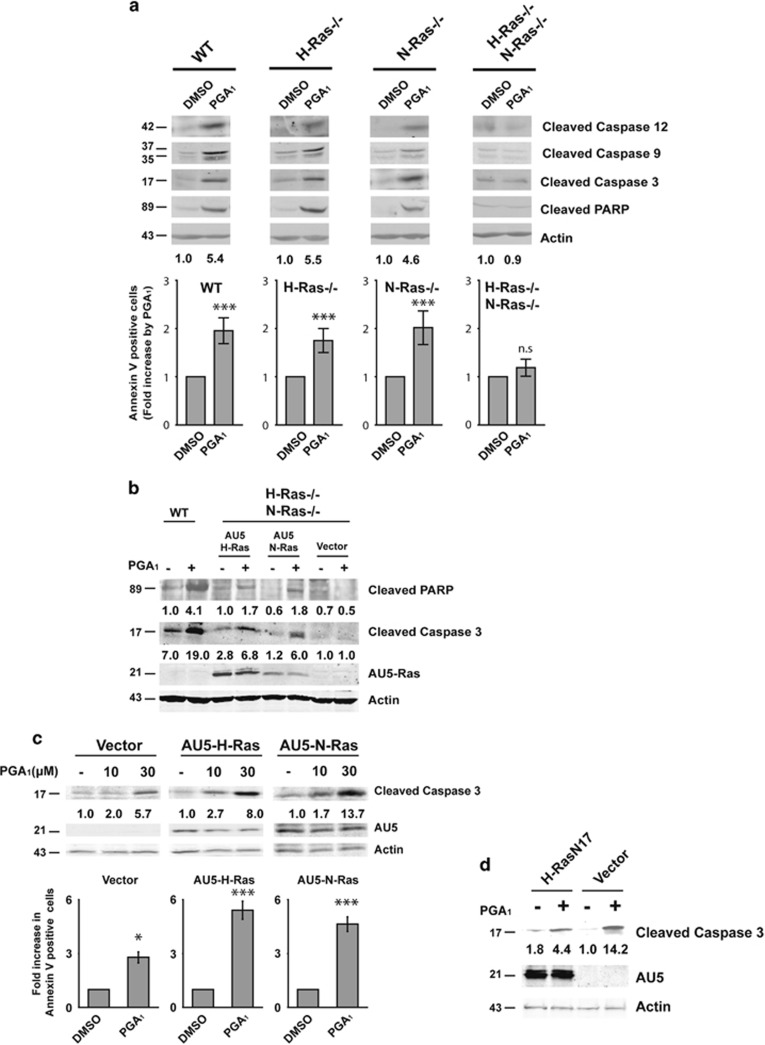
Apoptosis induced by PGA_1_ depends of H-Ras and N-Ras. Serum-starved wt, H-Ras^−/−^, N-Ras^−/−^, or H-Ras^−/−^/N-Ras^−/−^ MEFs were treated as in Figure 1b. (**a**, upper panel) A representative analysis as in Figure 1b. (Lower panel) Annexin V/FITC analysis was performed as in Figure 1c. Results (mean±S.D., *n*=4) are expressed as fold increase in the percentage of DMSO-treated cells ***P≤0.001, NS, nonsignificant. (**b**) H-Ras^−/−^/N-Ras^−/−^ MEFs transiently transfected with pCEFL-KZ-AU5-H-Ras-wt (AU5-H-Ras), pCEFL-KZ-AU5-N-Ras-wt (AU5-N-Ras), or pCEFL-KZ-AU5 (Vector) were treated as in (**a**). Wild-type MEFs (WT) were analyzed in parallel using the same stimuli. Expression level of the ectopic AU5-Ras was assessed using anti-AU5. Levels of cleaved PARP (determined by densitometry) are provided at the bottom of the panel. (**c**) NIH3T3 cells stably transfected with pCEFL-KZ-AU5-H-Ras-wt (AU5-H-Ras), pCEFL-KZ-AU5-N-Ras-wt (AU5-N-Ras), or pCEFL-KZ-AU5 (Vector) were serum starved for 24 h and then incubated with vehicle (−) or PGA_1_ (10 and 30 *μ*M) and harvested 3 h after treatment. (Upper panel) Cell lysates were analyzed as in (**b**). (Lower panel) Annexin V/FITC staining analysis was performed as above (using 30 *μ*M of PGA_1_). Results (mean±S.D., *n*=3) are expressed as fold increase in the percentage of DMSO-treated cells. ***P≤0.001 and *P≤0.05 *versus* non-stimulated (DMSO) cells. (**d**) NIH3T3 cells transiently transfected with pCEFL-KZ-AU5-H-Ras-N17 (H-Ras-N17) or pCEFL-KZ-AU5 (Vector) were serum starved for 24 h and then incubated with vehicle (−) or 30*μ*M PGA_1_ (+). Cell lysates were obtained 3 h after treatment and analyzed as in (**c**). The levels of cleaved caspase-3 (**a**–**d**), which were determined by densitometry, are provided at the bottom of each panel. The figures indicate the average levels of cleaved caspase-3 relative to controls of at least four independent experiments (S.D. <10% average in each case)

PGA_1_-induced apoptosis has been reported in several cellular models, including cancer cells.^10^ However, the precise molecular mechanism by which PGA_1_ elicits cell elimination remains unclear. We have previously reported that PGA_1_ binds to and activates H- Ras, N-Ras, and K-Ras to a similar extent.^20^ As Ras proteins promote activation of the effector cascades that trigger apoptosis, we used a knockout approach to evaluate the potential role of these proteins in mediating PGA_1_-dependent apoptosis (Supplementary Figure S2A). Analysis of relevant apoptosis markers upon PGA_1_ incubation in mouse embryonic fibroblasts (MEFs) for H-Ras^−/−^ or N-Ras^−/−^ did not reveal differences in caspase activation or in PARP proteolysis compared with wt MEFs. Interestingly, in H-Ras^−/−^/N-Ras^−/−^ double-knockout MEFs, PGA_1_ failed to induce caspase activation and apoptosis (Figure 2a). Ectopic restitution of H-Ras or N-Ras expression in double-knockout cells caused cell death increase, activation of PARP, and caspase-3 by PGA_1_ (Figure 2b and Supplementary Figure S2B). Given that PGA_1_-induced apoptosis requires expression of H-Ras or N-Ras, we reasoned that overexpression of these isoforms could synergize with the PGA_1_ effects. Indeed, PGA_1_-induced dose-dependent increases in cleaved caspase-3 levels were much greater in NIH3T3 cell lines stably overexpressing ectopic AU5-H-Ras-wt or AU5-N-Ras-wt than in control cell lines (Figure 2c, upper panel). In agreement with these data, treatment of Ras-overexpressing fibroblasts with PGA_1_ markedly increased the population of annexin V-positive cells (Figure 2c, lower panel). Moreover, ectopic expression of a dominant-negative mutant of H-Ras (H-Ras-N17) reduced caspase-3 activation levels upon stimulation with PGA_1_ (Figure 2d). Taken together, these results show that signaling by H-Ras and N-Ras, but not K-Ras, are required to promote PGA_1_-induced apoptosis.

### Ras-Cys118 residue is required for PGA_1_-induced apoptosis

**Figure 3:**
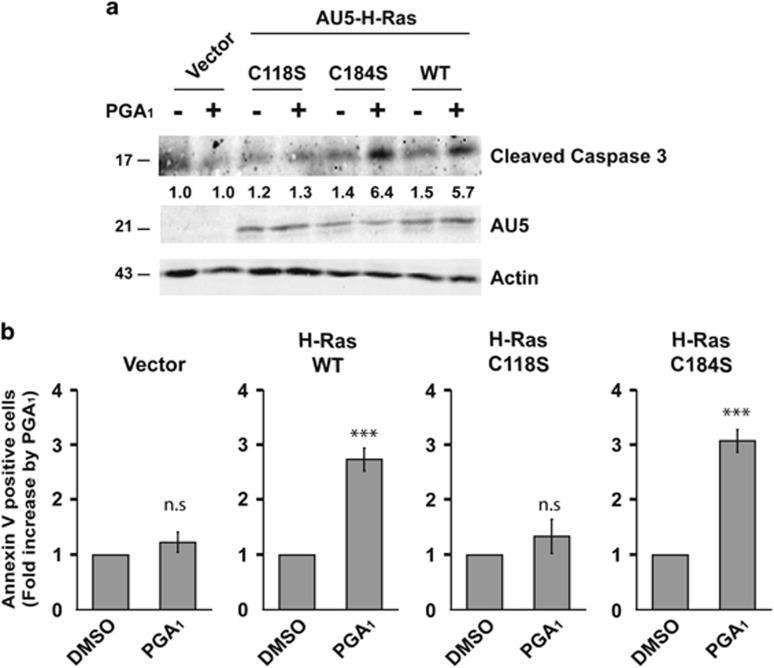
Ras-Cys118 is necessary for PGA_1_-induced apoptosis. H-Ras^−/−^/N-Ras^−/−^MEFs transiently transfected with pCEFL-KZ-AU5 (Vector), pCEFL-KZ-AU5-H-Ras-wt (WT), pCEFL-KZ-AU5-H-Ras-C118S (C118S), or pCEFL-KZ-AU5-H-Ras-C184S (C184S) were treated as in Figure 2b. (**a**) Cell lysates were as in Figure 2b. Levels of cleaved caspase-3 are provided at the bottom (S.D. <10% average in each case, *n*=4). (**b**) Annexin V/FITC staining analysis was performed as in Figure 2c (mean±S.D., *n*=4). ***P≤0.001 *versus* non-stimulated (DMSO) cells

Our previous studies showed that PGA_1_ binds preferably to Cys118 in all three Ras proteins, and Cys184 and Cys181 in H-Ras. However, rather than Cys184 or Cys181, PGA_1_ activates H-Ras by binding to Cys118.^20^ As Cys181 is shared by N-Ras and H-Ras, whereas Cys184 is unique to H-Ras, we speculated that Ras-Cys118 was the main target for PGA_1_ in mediating apoptosis. To determine the relative contribution of Cys118, double-knockout MEFs were transfected with H-Ras-wt, H-Ras-C118S, or H-Ras-C184S. As shown in Figure 3a and Supplementary Figure S2B, expression of H-Ras-wt or H-Ras-C184S induced cell death increase and caspase-3 activation in response to PGA_1_, whereas expression of H-Ras-C118S did not. Furthermore, analysis of transfected PGA_1_-stimulated MEFs showed that the C118S mutant did not increase the annexin V-positive cell population (Figure 3b). These data provide strong evidence that Cys118 of H- and N-Ras is necessary for PGA_1_-induced apoptosis.

### PGA_1_ induces Ras activation in endomembranes

**Figure 4:**
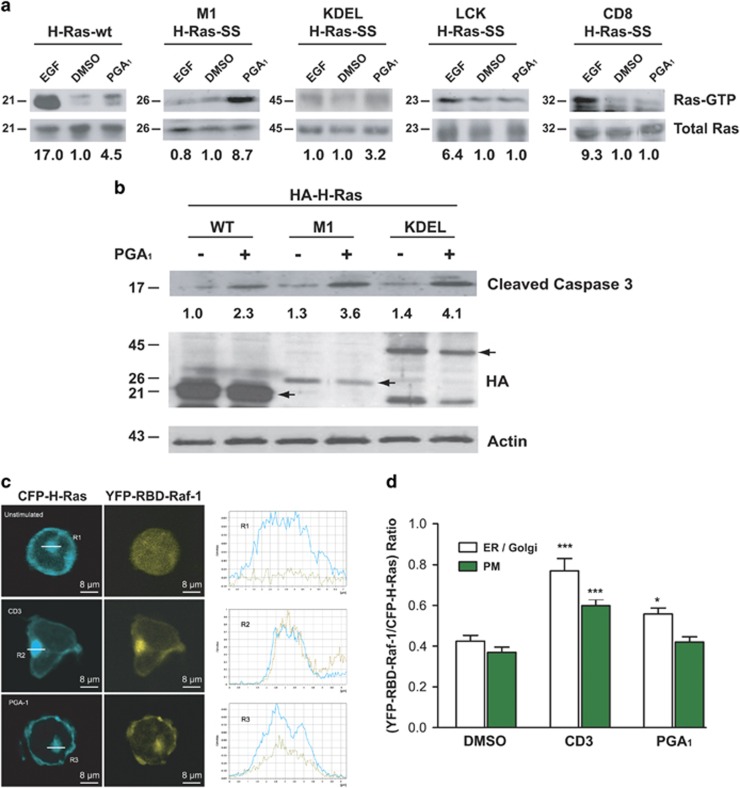
Activation of Ras by PGA_1_ occurs in endomembrane compartments. (**a**) HeLa cells transiently transfected with pCEFL-KZ-AU5, pCEFL-KZ-HA-H-Ras-wt, pCEFL-KZ-HA-M1-H-Ras-SS, pCEFL-KZ-HA-KDEL-H-Ras-SS, pCEFL-KZ-HA-LCK-H-Ras-SS, or pCEFL-KZ-HA-CD8-H-Ras-SS were serum starved for 24 h and then incubated with DMSO, EGF (100 ng/ml, for 15 min) or PGA_1_ (30 *μ*M for 15 min). Ras-GTP was recovered from cell lysates by binding to immobilized GST containing the Ras-GTP binding domain of RAF and detected by immunoblotting with anti-HA monoclonal antibody (upper panel). The expression levels of the transfected HA-H-Ras were detected by immunoblotting the cell extracts with the corresponding anti-HA monoclonal antibody (lower panel). Ras-GTP levels were determined by densitometry and are shown at the bottom (S.D. <10% average in each case, *n*=4). (**b**) H-Ras^−/−^/N-Ras^−/−^ MEFs transiently transfected with pCEFL-KZ-HA-H-Ras-wt (WT), pCEFL-KZ-HA-M1-H-Ras-SS (M1), or pCEFL-KZ-HA-KDEL-H-Ras-SS (KDEL) were treated and analyzed as in Figure 2b. Levels of cleaved caspase-3 are provided at the (S.D. <10% average in each case, *n*=3); black arrows show transfected Ras. (**c**) CH7C17 cells transiently co-transfected with pEYFP-Raf-RBD (yellow, RBD-Raf-1) and pECFP-H-Ras-wt (cyan, H-Ras-CFP) were serum starved for 24 h and then incubated with DMSO (non-stimulated), 30 *μ*M PGA_1_, or 5 *μ*g/ml anti-CD3 Ab. The subcellular localization of RBD-Raf-1 and H-Ras was recorded using confocal fluorescence microscopy at 15 min after treatment. Right, the corresponding profile analysis of the intensity and distribution of H-Ras (cyan solid line) and RBD-Raf-1 (yellow dashed line) along a cross-section of the regions of interest R1–3 (ER/Golgi) are shown. (**d**) Quantitative analysis of the cellular localization of YFP-RBD-Raf-1 (RBD) in CH7C17 cells transiently co-transfected with pEYFP-Raf-RBD and pECFP-H-Ras-wt. Histograms show the ratio of YFP-RBD-Raf-1/CFP-H-Ras accumulation in the ER/Golgi complex (white) or at the plasma membrane (PM) (green) of CH7C17 cells. At least 100 cells were scored for each condition. Data are expressed as mean±S.D. (*n*=3). ***P≤0.001 and *P≤0.05 *versus* non-stimulated (DMSO) cells (−)

To examine whether activation of Ras by PGA_1_ could be affected by intracellular localization, we measured the Ras-GTP levels induced by PGA_1_ in HeLa cells transiently transfected with H-Ras targeted either to the plasma membrane or to endomembrane compartments. We found that M1-H-Ras-SS and, to a lesser extent, KDEL-H-Ras-SS, H-Ras constructs targeted to the ER and the Golgi apparatus, respectively,^21^ were activated after stimulation by PGA_1_ (Figure 4a). In contrast, LCK-H-Ras-SS and CD8-H-Ras-SS, which directed H-Ras specifically towards the plasma membrane (lipid rafts and bulk membrane, respectively^21^) were not activated by PGA_1_. Moreover, as shown in Figure 4b (and Supplementary Figure S3A), activated caspase-3 and cell death was detected in double-knockout MEFs transfected with ER-targeted form M1-H-Ras-SS.

To confirm that PGA_1_ preferentially activated Ras on endomembranes, we studied the subcellular localization of PGA_1_-induced Ras activation *in vivo* using double fluorescence confocal microscopy analysis of CH7C17 Jurkat cells, a human T-cell line that does not express H-Ras, but activates endogenous N-Ras and ERK in response to PGA_1_ or CD3 and apoptosis in response to PGA_1_ (Supplementary Figures S3B–D). For this purpose, we co-transfected CH7C17 cells with enhanced cyan fluorescent protein (CFP)-H-Ras, which distributes between the plasma membrane and the ER/Golgi complex, and YFP-RBD-Raf-1, a yellow fluorescent tracker of H-Ras activation in live cells. Whereas YFP-RBD-Raf-1 was localized throughout the cytoplasm of non-stimulated cells, stimulation with CD3 or PGA_1_ increased colocalization of YFP-RBD-Raf-1 with CFP-H-Ras on endomembranes (Figure 4c) and was higher than on the plasma membrane (Figure 4d). Thus, these results strongly suggest that PGA_1_ activates Ras proteins in endomembrane compartments.

### PGA_1_ induces calpain activation in fibroblasts

**Figure 5:**
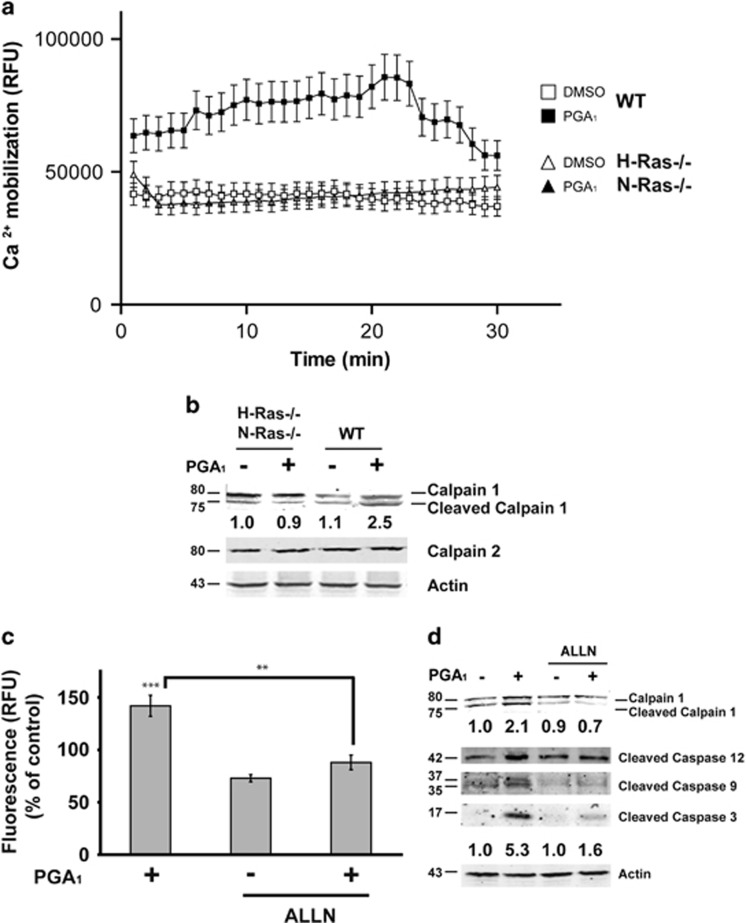
PGA_1_ induces calpain activation. Serum-starved wt or H-Ras^−/−^/N-Ras^−/−^ MEFs were stimulated with 30 *μ*M PGA_1_ or DMSO. (**a**) Increase in cytosolic Ca^2+^ levels. Measurements are given in relative fluorescent units (RFU) during 30 min. Data are expressed as mean±S.D. of three independent experiments. (**b**) Cell lysates were obtained 3 h after treatment and analyzed for detection of calpain-1, calpain-2, and actin. (**c**) Calpain activity was measured 3 h after treatment. Measurements are given in RFUs. Data are expressed as mean±S.D. (*n*=3). ***P≤0.001 *versus* control and **P≤0.01. (**d**) Serum-starved wt MEFs were stimulated with 30 *μ*M PGA_1_ or DMSO in the presence of the calpain inhibitor ALLN (10 *μ*M). Cell lysates were obtained 3 h after treatment and analyzed as in Figure 2b and in (**b**). Levels of cleaved calpain-1 and caspase-3 are denoted at the bottom of each panel (S.D. <10% average in each case, *n*=3)

Caspase-12 has been localized on the cytoplasmic side of the ER, and is activated by alterations of the ER homeostasis such as mobilization of intracellular calcium. As caspase-12 is activated in response to PGA_1_ stimulation and the activation of caspase-12 may occur through ER stress-induced calpains,^22^ we tested the possible involvement of these endopeptidases on the PGA_1_-induced apoptosis. Figure 5a shows a weak but sustained increase of intracellular calcium concentration upon PGA_1_ treatment in wt MEFs; in contrast, the calcium levels displayed in H-Ras^−/−^/N-Ras^−/−^ double-knockout MEF cells were not significantly elevated. Western blotting analysis of PGA_1_-treated wt MEFs revealed that the levels of autoproteolytically cleaved calpain-1 were much greater than in H-Ras^−/−^/N-Ras^−/−^double-knockout MEFs, whereas the levels of calpain-2 remained unaltered (Figure 5b). Furthermore, ALLN ((2*S*)-2-acetamido-4-methyl-*N*-((2*S*)-4-methyl-1-oxo-1-(((2*S*)-1-oxohexan-2-yl)amino)pentan-2-yl)pentanamide), a calpain specific inhibitor, completely blocked the calpain activity induced by PGA_1_ in wt MEFs (Figure 5c) and totally inhibited the proteolysis of both caspase-12 and calpain-1 and, in a lower extent, the activation of caspase-9 and -3 (Figure 5d). Taken together, these data indicate that calpain activity is required for cleavage of caspase-12 induced by PGA_1_.

### ERK activation is required for PGA_1_-dependent apoptosis

**Figure 6:**
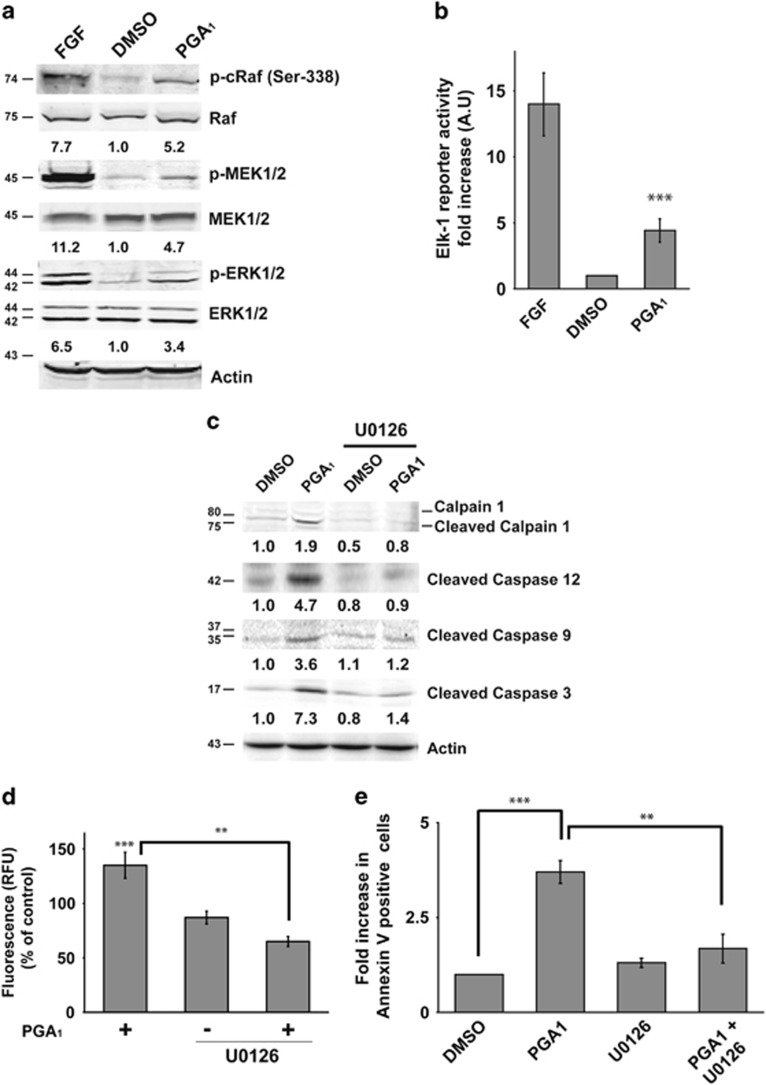
ERK activation is necessary for PGA_1_-dependent apoptosis. Serum- starved wt MEFs were stimulated with 30 *μ*M PGA_1_, FGF, or DMSO. (**a**) Lysates prepared from cells 15 min after treatment underwent western blotting for relevant components of the RAF-MEK-ERK pathway. Levels of p-c-RAF, p-MEK1/2, and p-ERK1/2 are provided at the bottom of each panel (S.D. <10% average in each case, *n*=4). (**b**) Cells were co-transfected with the plasmids pcDNAIII-Gal4-Elk-1, pGal4-Luc, and pTK-Renilla 24 h before synchronization. Firefly luciferase activity was determined 6 h after stimulation with PGA_1_ and normalized to Renilla luciferase activity. Results, in arbitrary units (AU), are expressed as mean±S.D. (*n*=3). ***P≤0.001 *versus* control. (**c**) MEK1/2 inhibitor U0126 (5 *μ*M) was added ~50 min before PGA_1_ treatment (30 *μ*M) and left in the medium until processing. Cell lysates were prepared and analyzed as in Figure 5c. Levels of cleaved calpain-1, cleaved caspase-12, cleaved caspase-9 and caspase-3 are indicated at the bottom of each panel (S.D. <10% average in each case, *n*=3). (**d**) Calpain activity was measured as in Figure 5d. Data are expressed as mean±S.D. (*n*=3). ***P≤0.001 *versus* control and **P≤0.01. (**e**) Annexin V/FITC staining analysis was performed as in Figure 1c. Results are expressed mean±S.D. (*n*=4). ***P≤0.001 and **P≤0.01

One of the most important signaling pathways downstream of Ras is the RAF-MEK-ERK cascade, and previous work has demonstrated that calpain is activated by ERK signaling.^23^ As PGA_1_ induced both activation of Ras and calpain proteins, we assessed the activation of these kinases downstream of Ras. Treatment of wt MEFs with PGA_1_ induced activation of RAF, MEK, and ERK, although to a lesser extent than FGF (Figure 6a). Consistent with these results, PGA_1_ treatment promoted significant activation of an Elk-1 luciferase reporter, although this activation was once again weaker than that induced by FGF (Figure 6b). Despite the weak and short duration of phospho-ERK levels (Supplementary Figures S4A and B), we speculated that this activation could be responsible for the apoptosis observed. To examine whether ERK activation was required for PGA_1_-induced apoptosis, the MEK1/2 inhibitor U0126 was added before PGA_1_ treatment (~50 min) and left until the end of PGA_1_ treatment. As shown in Figure 6c (and Supplementary Figure S4C), in wt MEFs the inhibitor completely blocked calpain-1 and caspase-12, -9 and -3 activation induced by PGA_1_. Moreover, both the calpain activity and Annexin V-positive cell number were markedly reduced in the presence of the MEK inhibitor upon PGA_1_ stimulation (Figures 6d and e). Taken together, these data strongly suggest that PGA_1_ promotes apoptosis through the activation of the RAF-MEK-ERK pathway.

### PGA_1_ also induces apoptosis in human cancer cells

**Figure 7:**
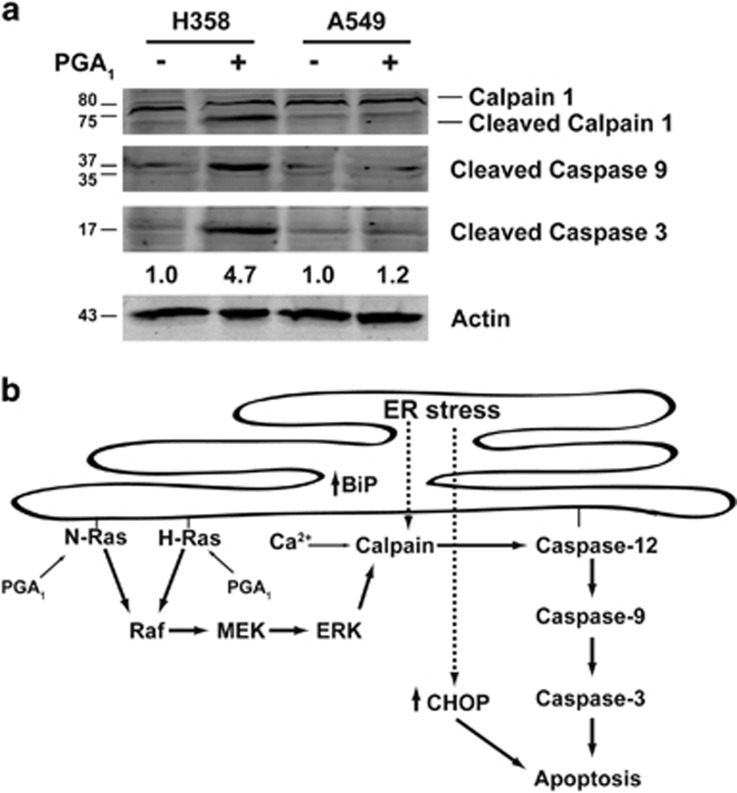
PGA_1_ induces cell death in cancer cells. (**a**) H358 and A549 cells were treated and analyzed in Figure 2a. Levels of cleaved caspase-3 are at the bottom (S.D. <10% average in each case, *n*=3). (**b**) A model illustrating the signaling cascades initiated by H-Ras and N- Ras activation after PGA_1_ and their roles in apoptosis

Apoptosis is the leading mechanism proposed to account for the antitumoral and antiproliferative effects of PGA_1_ in cancer cells.^24^ Indeed, we assayed PGA_1_-induced apoptosis in a panel of 14 human cell lines from different cancer types (Supplementary Figures S5A and S6). Although the sensitivity to PGA_1_ showed differences between the distinct cancer cell lines, it remained very similar among lines of the same tumor type, with the exception of the non-small-cell lung cancer (NSCLC) lines. Thus, in this case, most H358 and H23 cells underwent apoptosis upon PGA_1_ treatment, whereas A549, H522, and H2126 cells showed higher resistance to PGA_1_-induced cell death. Next, we examined whether PGA_1_ treatment activated in human cancer cells the same mechanism than in MEFs. Western blotting and analysis of apoptotic markers in H358 and A549, K-RasV12-mutated NSCLC lines with different sensitivity to PGA (Figure 7a), showed activation of calpain-1, caspase-9, and caspase-3 in H358 cells but not in A549 cells. Altogether, these data suggest that in human cancer cells the mechanism of PGA_1_-induced apoptosis may be mediated by a similar set of signaling proteins than in mouse embryo fibroblast cells.

## Discussion

Although PGA_1_ displays potent antiproliferative activity, a fundamental yet unanswered question is the underlined mechanism. The cyclopentane ring of CyPG posseses an α-, *β*-unsaturated carbonyl group that reacts with sulfhydryl groups in cysteine residues of cellular proteins by means of Michael's addition.^25^ During recent years, several protein targets have been identified for CyPG, including NF-*κ*B, AP1, Keap-1, and LKB1.^26, 27, 28^ We have reported that Ras proteins can also be modified by the addition of 15d-PGJ_2_ and PGA_1_; in fact, the three Ras isoforms (H-Ras, N-Ras, and K-Ras4B) bind to and are activated by PGA_1._^14, 20^ Induction of apoptosis by PGA_1_ occurs in different cell types, such as in endothelial cells, gastric epithelial cells, lung, prostate, and colon cancer cells.^16, 17, 29^ In contrast, some cellular models suggest that PGA_1_ protects against apoptosis through a mechanism that involves inhibition of NF-*κ*B activation.^30^ Our results show that PGA_1_ induces apoptosis by a caspase-dependent mechanism that requires either H-Ras or N-Ras, but not K-Ras. PGA_1_-induced apoptosis is likely initiated by an ER/Golgi complex pool of Ras and requires activation of ERK. Indeed, the only MEFs that showed activation of caspase-3 after stimulation with PGA_1_ were those that harbor the *H-ras* and/or the *N-ras* genes. Consistent with this observation, the ectopic overexpression of H-Ras or N-Ras in double-knockout MEFs was sufficient to rescue the apoptotic levels induced by PGA_1_. Moreover, NIH3T3 cells constitutively overexpressing ectopic H-Ras or N-Ras displayed synergistic enhancement of PGA_1_ apoptotic effects, even at low concentrations. In addition, overexpression of a dominant-negative mutant of H-Ras (H-Ras-N17) in fibroblasts caused a significant reduction in caspase-3 activation, indicating that functional Ras signaling is essential for PGA_1_-induced apoptosis.

In this study, we have provided evidence that the PGA_1_ activates Ras proteins in the ER and in the Golgi complex, but not at the plasma membrane (lipid rafts or bulk membrane). CFP-H-Ras in CH7C17 cells colocalized *in vivo* with YFP-RBD-Raf-1 in endomembrane systems upon stimulation with PGA_1_. A large body of evidence supports the possibility that H-Ras/N-Ras activation can occur in these organelles; nevertheless, the specific outcome of this activation remains debated. Thus, activated H-Ras targeting the Golgi apparatus has been reported to elicit strong activation of ERK and AKT, but weak activation of JNK, whereas the opposite effects were described when H-Ras targeted the ER.^31^ Other studies,^32^ however, which used the Golgi apparatus-tethering signal KDELr containing the mutation N193D that fixes it permanently to the Golgi apparatus, reported that activated H-Ras tethered to the Golgi apparatus (KDEL-H-RasV12-SS) only induced Ral and JNK signaling, whereas hyperactive H-Ras targeting the ER (M1-H-RasV12-SS) displayed a potent activation of ERK and AKT. We observed that PGA_1_ induces transitory and weak activation of ERK, although no effects on AKT or JNK phosphorylation were observed. An unsolved issue concerns the reason why Ras proteins resulted specifically activated by PGA_1_ in the ER and Golgi. The ability of PGA_1_ to stimulate transitory activation of ERK appears to contradict most studies about apoptosis induced by the Ras-RAF-MEK-ERK pathway, where in response to numerous stress stimuli, ERK activation was strong and sustained, whereas transient activation of ERK protects against death.^33^ However, overexpression of the hyperactive M1-H-RasV12-SS that promotes constitutive ERK activation and generates an antiapoptotic signal^32^ did not induce apoptosis in wt MEFs (Supplementary Figure S4D).

Further studies are necessary to elucidate the transduction pathway downstream of ERK that is associated with caspase-3 cleavage upon stimulation with PGA_1_. Nevertheless, it has been reported that growth factors activate calpains downstream of ERK.^34^ Our results further support a link between PGA_1_, ERK, and calpain activation, as the MEK inhibitor U0126 abolished ERK, calpain, and caspase activation and therefore PGA_1_-induced apoptosis, suggesting that calpains have an essential role in the apoptotic pathway induced by PGA_1_ downstream of ERK activation. Although activation of caspase-12, a marker of ER stress, may occur through ER stress-induced calpains,^35^ we do not know if the Ras activation induced by PGA_1_ in endomembranes could trigger an ER stress response. CyPGs exert a variety of biological actions, most remarkably among them the stress response. PGA_1_ is an important inducer of stress response by increasing the synthesis of HSPs and activating heat-shock transcription factors.^36^ In rats with acute liver damage, pretreatment with PGE_1_ and somatostatin inhibits apoptosis and alleviates ER stress by induction of HSP70 and BiP/GRP78 (immunoglobulin-binding protein/), but suppression of CHOP (C/EBP homologous protein).^37^ Indeed, Δ^12^-PGJ_2_ and PGA_1_ induce BiP gene expression through unfolded protein response (UPR).^38^ BiP is a molecular chaperone that is synthesized constitutively, although accumulation of unfolded proteins in the ER changes in Ca^2+^ levels, reducing environment and block of glycosylation induces transcription of the BiP gene.^39^ Furthermore, ER stress can be induced by a variety of cellular functions, including a wide spectrum of Ras targets. Thus, in primary human melanocytes H-RasV12-induced senescence was mediated by the UPR, initiated by the PI(3)K/AKT pathway, while in mouse fibroblasts or in established or immortalized cells, the UPR drive to cell cycle arrest.^40, 41^ Oncogenic H-Ras downregulates CHOP expression and is required for transformation while the expression of exogenous CHOP blocks transformation in NIH3T3 cells.^42^ Recent studies have demonstrated that the activation of K-Ras on the ER surface cooperates with Nox4 to initiate the UPR and autophagy and therefore prevent cell death and promote differentiation in HUVEC cells.^43^ We found evidences supporting the existence of a PGA_1_-induced UPR. Indeed, upon prostaglandin stimulation, we observed an elevation in the levels of the proteins BiP and CHOP in wt MEFs and H358 cells (Supplementary Figures S5B and S5C). These data strongly suggest that PGA_1_ could trigger an ER stress response through H- and N-Ras activation. In summary, our study provides new insights into the molecular mechanism underlying the induction of apoptosis by PGA_1_. Our findings reveal a novel mechanism by which PGA_1_ stimulates cell death as a consequence of specific activation of H-Ras and N-Ras in endomembranes (ER and the Golgi complex), whereas K-Ras was dispensable for this function (summarized in Figure 7b). These data also have implications for signal transduction and cancer, as the RAF-MEK-ERK cascade is the signaling pathway responsible for this cell death through ERK-induced calpain activity and subsequent caspase activation even in cells with deletion of the *TP53* gene and that harbor oncogenic K-Ras as H358 cells. Our results highlight the relevance for physiological outcomes of the intensity, duration, and specific subcellular location of Ras activation. PGA_1_ and some analogs received great attention in the 1990 s; although some analogs showed promising results in preclinical studies, their use in the clinical practice has not been put forward.^24^ More recently, other potential clinical uses of PGA_1_ or its analogs have been explored, including the potential beneficial effects of A-type cyclopentenone prostaglandins in atherosclerosis^44^ and its potential use in the inhibition of the aldo-keto reductase enzymes, which are involved in cancer resistance to chemical treatment and in the development of diabetic complications.^45, 46^ Here our data highlight the possibility that cyclopentenone prostaglandins, because of their specific cellular effects, can be useful in the design of new therapeutic drugs and these studies are under way.

## Materials and Methods

### Materials

Cell culture medium was purchased from Invitrogen (Carlsbad, CA, USA). The pancaspase inhibitors Z-Asp-CH2-DCB (Z-Asp-2,6-dichlorobenzoyloxymethylketone), U0126, and GW5074 were from Biomol Research Laboratories Inc. (Plymouth Meeting, PA, USA). PGA_1_, propidium iodide, DAPI, and recombinant bFGF were from Sigma-Aldrich (St. Louis, MO, USA). DEA-NO was from Alexis Biochemicals (Carlsbad, CA, USA).

### Cell culture

HeLa, SH-SY5Y, U373, TPC1, A2780, SCC12, A431, SW480 (ADH-V and S: normal or overexpressing Spry2),^47^ HT29, A549, H522, H2126, H358, H23, and NIH3T3 cells were obtained from American Type Culture Collection (ATCC, Manassas, VA, USA) and cultured in DMEM or RPMI medium supplemented with 10% FBS (Invitrogen), penicillin (100 U/ml), streptomycin (100 *μ*g/ml), and l-glutamine (2 mM) at 37 °C in a humidified 5% CO_2_ atmosphere. Knockout MEFs for *H-ras* and/or *N-ras* genes are described elsewhere.^48^ CH7C17 cells were cultured in RPMI medium supplemented with 10% FBS (Invitrogen). The synchronization process involved cells being cultured in normal medium for 24 h, serum starved for 24 h, and then treated with PGA_1_ in the absence of serum. Human cell lines were authenticated by the Genomics Service of Instituto de Investigaciones Biomédicas Alberto Sols (Madrid, Spain) using the *GenePrint* 10 System (Promega), which allows coamplification and three-color detection of 10 human *loci*: TH01, TPOX, vWA, amelogenin, CSF1PO, D16S539, D7S820, D13S317, D21S11, and D5S818. These *loci* collectively provide a genetic profile with a random match probability of 1 in 2.92 × 10^9^ and are used for human cell line and tissue authentication and identification and human cell line cross-contamination determination. STR profiles are sent for comparison against cell line databases such as ATCC and DSMZ (Deutsche Sammlung von Mikrorganismen and Zellkulturen). Cells were tested routinely to ensure there was no mycoplasma contamination (Universal Mycoplasma Detection Kit; ATCC, Manassas, VA, USA; no. 30-1012).

### Cell proliferation and cell cycle analysis

Cells (2.5 × 10^5^) were seeded in triplicate in 60 mm dishes and synchronized as described above. They were then treated with FGF, PGA_1_, or vehicle (dimethyl sulfoxide (DMSO)) for the specified times, trypsinized at different intervals, and counted with a hemocytometer. Images were collected with a DS-L1 Digital Sight Camera System (Nikon, Nikon Instruments Europe B.V., L'Hospitalet de Llobregat, Spain) coupled to a Eclipse TS100 inverted microscope, × 40 objective lens (Nikon). Cell cycle profiles were determined by propidium iodide staining (0.1 mg/ml) and flow cytometry.

### Annexin V-FITC staining

Cell lines were grown and treated as described above. Apoptosis was determined using fluorescein isothiocyanate-conjugated Annexin V (Annexin V-FITC)/Propidium Iodide Apoptosis Detection Kit (R&D systems, Minneapolis, MN, USA) according to the manufacturer's instructions. Cells that were positive for Annexin V-FITC and/or propidium iodide were analyzed using a BD FACS flow cytometer (SpainCustomer Service, San Agustin de Guadalix, Spain).

### Apoptosis assays

Cells were trypsinized, mounted on glass slides, and then fixed with 70% ethanol. Morphological changes in chromatin structure were assessed after staining with DAPI. Apoptosis was characterized according to chromatin condensation and fragmentation using fluorescence microscopy. The incidence of apoptosis in each preparation was analyzed by counting 500 cells and determining the percentage of apoptotic cells. Images were collected with an HCX PL APO × 40 NA 1.32 oil-immersion objective lens (Leica, Leica Microsistemas S.L.U., Barcelona, Spain).

### DNA constructs

The plasmids used pCEFL-KZ-AU5, pCEFL-KZ-AU5-H-Ras-wt, pCEFL-KZ-HA-H-Ras-wt, pCEFL-KZ-AU5-N-Ras-wt, and pGEX-GST-Raf-RBD have been described elsewhere,^14, 49, 50, 51, 52^ as have pCEFL-KZ-AU5-H-Ras-C118S (H-Ras-C118S), pCEFL-KZ-AU5-H-Ras-C184S pCEFL-KZ-HA-M1-H-Ras-SS, pCEFL- KZ-HA-KDEL-H-Ras-SS, pCEFL-KZ-HA-CD8-H-Ras-SS, pCEFL-KZ-HA-LCK-H-Ras-SS, pCEFL-KZ-HA-M1-H-RasV12-SS, and pCEFL-KZ-HA-KDEL-H-RasV12-SS^21, 32^ and pEYFP-Raf-RBD and pECFP-H-Ras-wt.^53^

### Cell transfection studies

Transient transfection was performed in NIH3T3, HeLa, and MEFs using Jet-PeiTM (Polyplus-Transfection, Illkirch, France). CH7C17 cells (15 × 10^6^) were transfected using the Pulser X-cell Electroporation System (Bio-Rad, Hercules, CA, USA) at 250 V and 1200 *μ*F, with 25 *μ*g of expression plasmids encoding the indicated constructs.

### Reporter gene analysis

Cells were transfected with 16 ng of pCDNAIII-Gal4-Elk-1, 0.1 *μ*g of pRL-TK (a plasmid containing the Renilla luciferase gene under control of the HSV-TK promoter), and 0.3 *μ*g of the reporter plasmid pGal4-Luc (containing the Photinus luciferase gene controlled by six copies of a Gal4-responsive element).

### T-cell activation and confocal microscopy

CH7C17 cells (15 × 10^6^) were allowed to settle in LabTek II chambers (Nalge Nunc International, Sigma-Aldrich Quimica SL, Madrid, Spain) and maintained at 37 °C in a 5% CO_2_ atmosphere in phenol red-free RPMI medium 1640 containing 25 mM HEPES and 2% FBS in an incubator coupled to a Leica TCS SP2 confocal microscope (Leica Microsistemas S.L.U.). T cells transiently co-transfected with YFP-RBD-Raf-1 and CFP-H-Ras were serum starved at 37 °C for 2 h before stimulation with DMSO, PGA_1_ (30 *μ*M/15 min), or anti-human CD3*ɛ* T3b mAb (5 *μ*g/ml). Time-lapse confocal images were collected with an HCX PL APO × 40 NA 1.32 oil-immersion objective lens (Leica), and fluorescence images were captured every 1 min. Six confocal Z-sections were necessary to capture the entire fluorescent signal at each time. The ratio of fluorescence intensity between the cellular regions of interest was calculated in a single Z plane, corresponding with the maximum fluorescence plane, by using Leica Confocal Software, version 2.61 (Leica Microsystems, Leica Microsistemas S.L.U.). At least 100 cells were analyzed for each sample. For fluorescence profile analysis, 8 *μ*m cross-sections were drawn (white bar).

### Western blot analysis

Cells were lysed in a buffer containing HEPES 25 mM, pH 7.5, 150 mM NaCl, 1% NP40, 10% glycerol, 0.1% SDS, 1% sodium deoxycholate, 10 mM MgCl_2_, 2 mM EDTA and Protease Inhibitor Cocktail from Sigma-Aldrich (catalog number P8340). Cell extracts (20 *μ*g protein per lane) underwent SDS-PAGE before being transferred to PVDF membranes (Millipore, Bedford, MA, USA). After blocking with 5% milk in 0.1% Tween-20/PBS, membranes were incubated with the primary antibody. The primary antibodies used at 1 : 1000 dilution were caspase-3 (8G10) (Cell Signaling Technology (CST), Boston, MA, USA; catalog number 9665), caspase-9 (CST; catalog number 9508), caspase-12 (CST; catalog number v2202), caspase-8 (CST; catalog number 4927), PARP (CST; catalog number 9542), p-c-Raf (Ser 338) (CST; catalog number 9427), MEK (CST; catalog number 9122), p-MEK (Ser217/221)(CST; catalog number 9121), ERK (CST; catalog number 4696), p-ERK (thr202/tyr204) (CST; catalog number 9101), calpain-1 (CST; catalog number 2556), calpain-2 (CST; catalog number 2539), BiP (CST; catalog number 3138), and CHOP (CST; catalog number 2895), K-Ras (Santa Cruz Biotechnology Inc., Santa Cruz, CA, USA; catalog number sc-30), N-Ras (sc-31), and CD3*ɛ* T3 mAb have been previously described.^49^ Actin (sc-47778), H-Ras (Y132) (Abcam, Cambridge, UK; catalog number ab32417), c-Raf (BD Transduction Laboratories; San Jose, CA, USA), HA (MMS-101R), and AU5 (MMS-135R) monoclonal antibodies (Berkeley Antibody Company, Berkeley, CA, USA), and anti-rabbit and anti-mouse (Li-Cor Biosciences, Lincoln, NE, USA). Anti-mouse or anti-rabbit horseradish peroxidase (1 : 3000; Bio-Rad) was used as a secondary antibody. Bands were visualized using an Enhanced Chemiluminescence Detection Kit (Amersham, Arlington Heights, IL, USA).

### Ras-GTP detection

Ras-GTP levels were estimated by pull-down assays.^14^

### DNA laddering

Floating and adherent cells were collected in TE/Triton buffer (0.2% Triton X-100, 10 mM Tris-HCl, pH 8.0, and 1 mM EDTA). Following incubation on ice for 10 min, an aliquot of the lysate containing total DNA was removed, and the remaining lysates were centrifuged for 15 min at 14 000 × *g* and 4 °C. Supernatant containing low-molecular-weight DNA was transferred to a fresh tube and treated with DNase-free RNase A (60 *μ*g/ml) for 1 h at 37 °C. SDS (0.5%) and proteinase K (150 *μ*g/ml) were added, and the samples were incubated for 1 h at 50 °C. DNA was precipitated by the addition of 0.1 volumes of 0.5 M NaCl and 1.0 volume of isopropanol, followed by incubation on ice for 10 min. After centrifugation, DNA in TE buffer (10 mM Tris-HCl, pH 8.0, 1 mM EDTA) was analyzed on 2% agarose gels.

### Intracellular calcium measurement

Measurement of intracellular calcium ions was determined by the Fluo-4 Direct Calcium Assay Kit (Invitrogen, Molecular Probes, Life Technologies S.A., Alcobendas, Spain) according to the manufacturer's instructions. Excitation at 494 nm and emission at 516 nm.

### Calpain activity determination

Detection of calpain activity was performed as described previously.^54^

### Statistical analysis

Data were analyzed using the SPSS software (SPSS Inc., Chicago, IL, USA). ANOVA was used to test statistical significance. Results are expressed as mean±S.D. of the indicated number of experiments. Statistical significance was evaluated using the *t*-test for unpaired observations. Western blots were analyzed using linear correlations between increasing amounts of protein and signal intensity.

## Abbreviations

15d-PGJ2: 15-deoxy-12,14-prostaglandin J2
ALLN: (2*S*)-2-acetamido-4-methyl-*N*-((2*S*)-4-methyl-1-oxo-1-(((2S)-1-oxohexan-2-yl)amino)pentan-2-yl)pentanamide
BiP: immunoglobulin-binding protein
CHOP: C/EBP homologous protein
DAPI: 4′, 6-diamidino-2-phenylindole
DMSO: dimethyl sulfoxide
ER: endoplasmic reticulum
FGF: fibroblast growth factor
GEF: guanine nucleotide-exchange factor
FITC: fluorescein isothiocyanate-conjugated
GDP: guanosine diphosphate
GTP: guanosine triphosphate
HSP: heat-shock protein
MEF: mouse embryonic fibroblast
NSCLC: non-small-cell lung cancer
PGA_1_: cyclopentenone prostaglandin A1
PARP: poly(ADP-ribose) polymerase
PPAR: proliferator-activated receptor
UPR: unfolded protein response
Z-Asp-CH2-DCB: Z-Asp-2,6-dichlorobenzoyloxymethylketone

## Supplementary Material

Supplementary Figure Legends

Supplementary Figure Add S1

Supplementary Figure Add S2
