# Supplementary figures and images for: PGA1-induced apoptosis involves specific activation of H-Ras and N-Ras in cellular endomembranes
Source: Cell Death Dis. 2016 Jul 28;7(7):e2311–. doi: 10.1038/cddis.2016.219 (PMC4973357; doi:10.1038/cddis.2016.219)

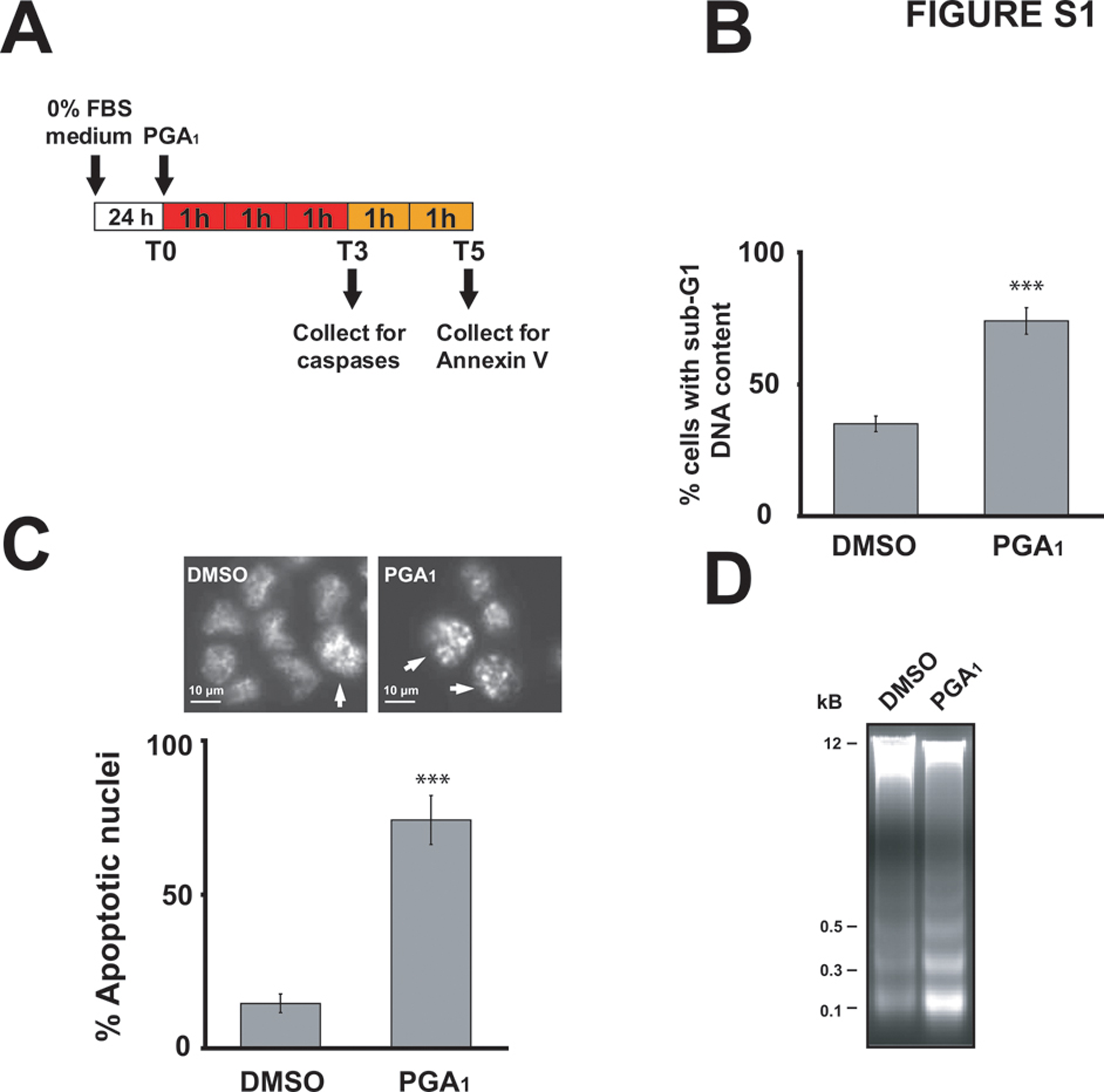

Supplement: Supplementary Figure S1 [file cddis2016219x1.tif]

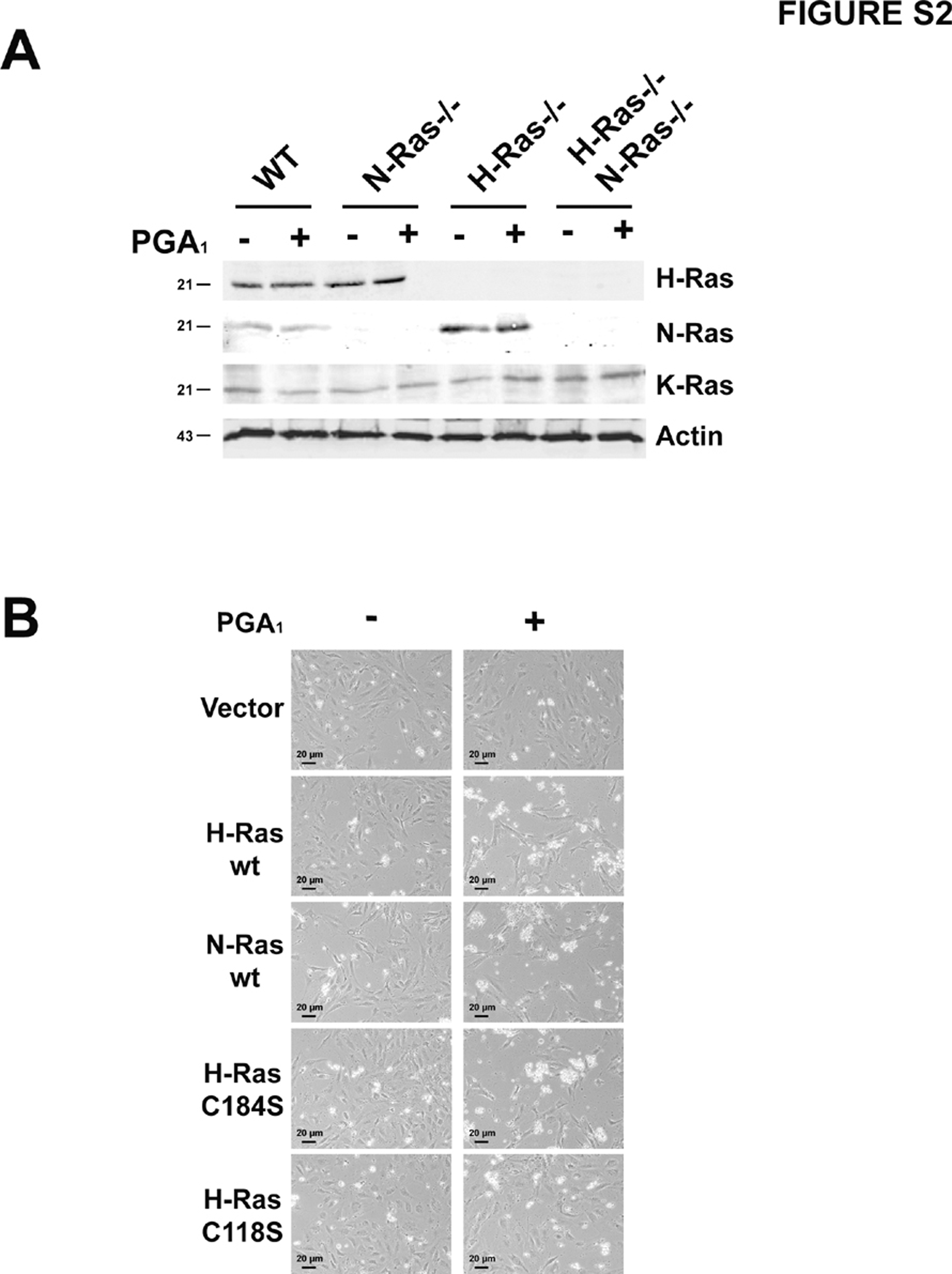

Supplement: Supplementary Figure S2 [file cddis2016219x2.tif]

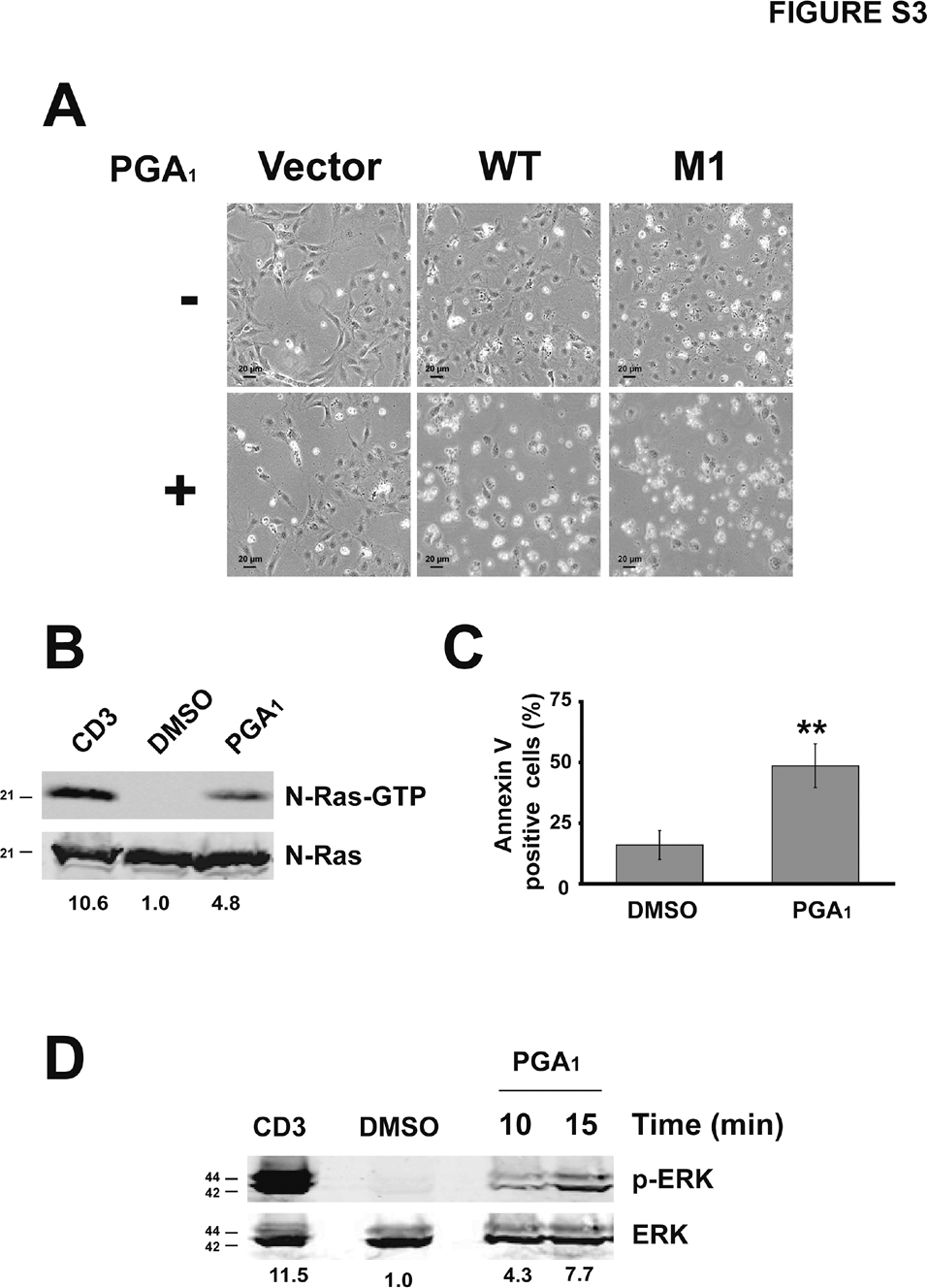

Supplement: Supplementary Figure S3 [file cddis2016219x3.tif]

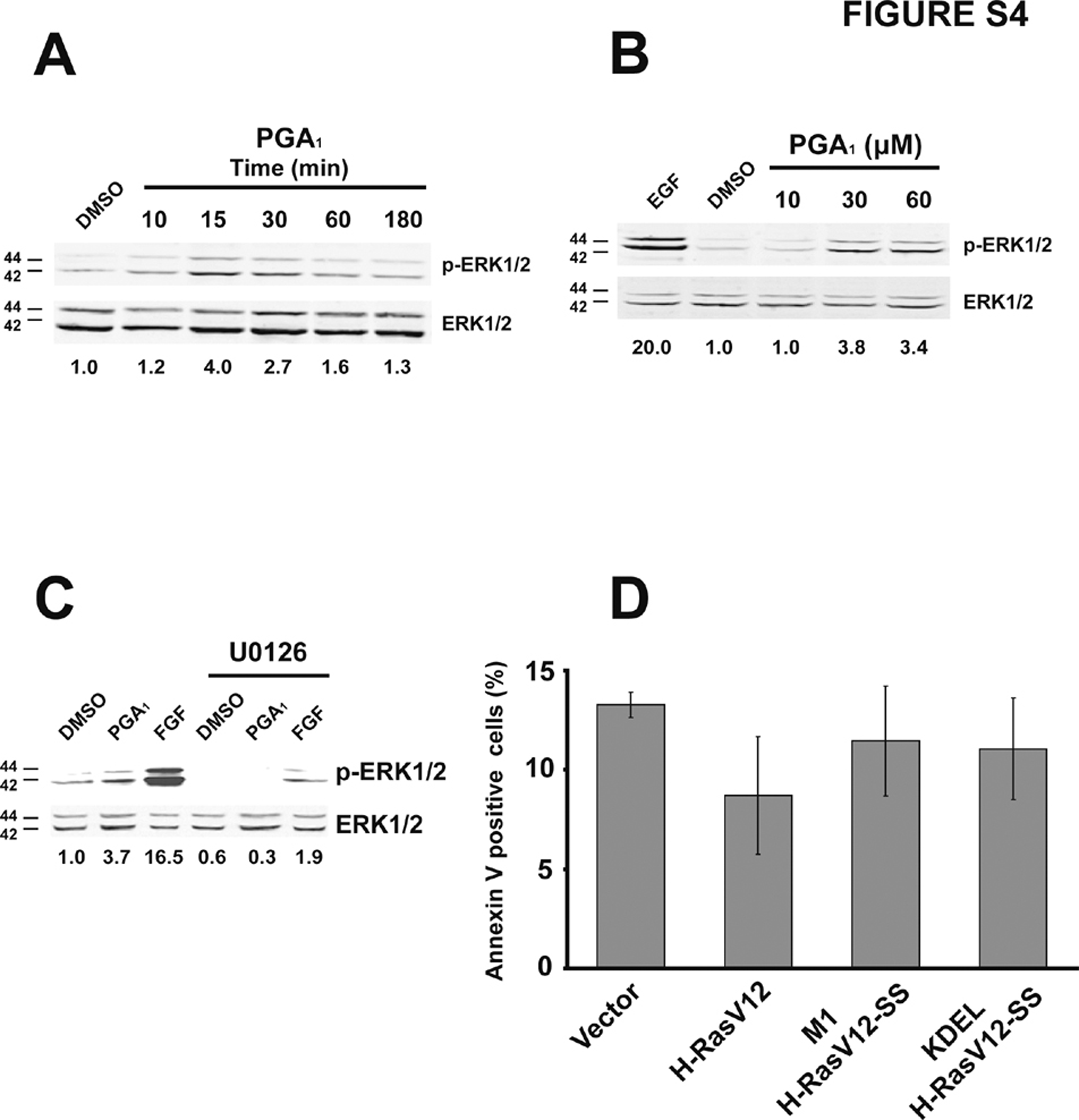

Supplement: Supplementary Figure S4 [file cddis2016219x4.tif]

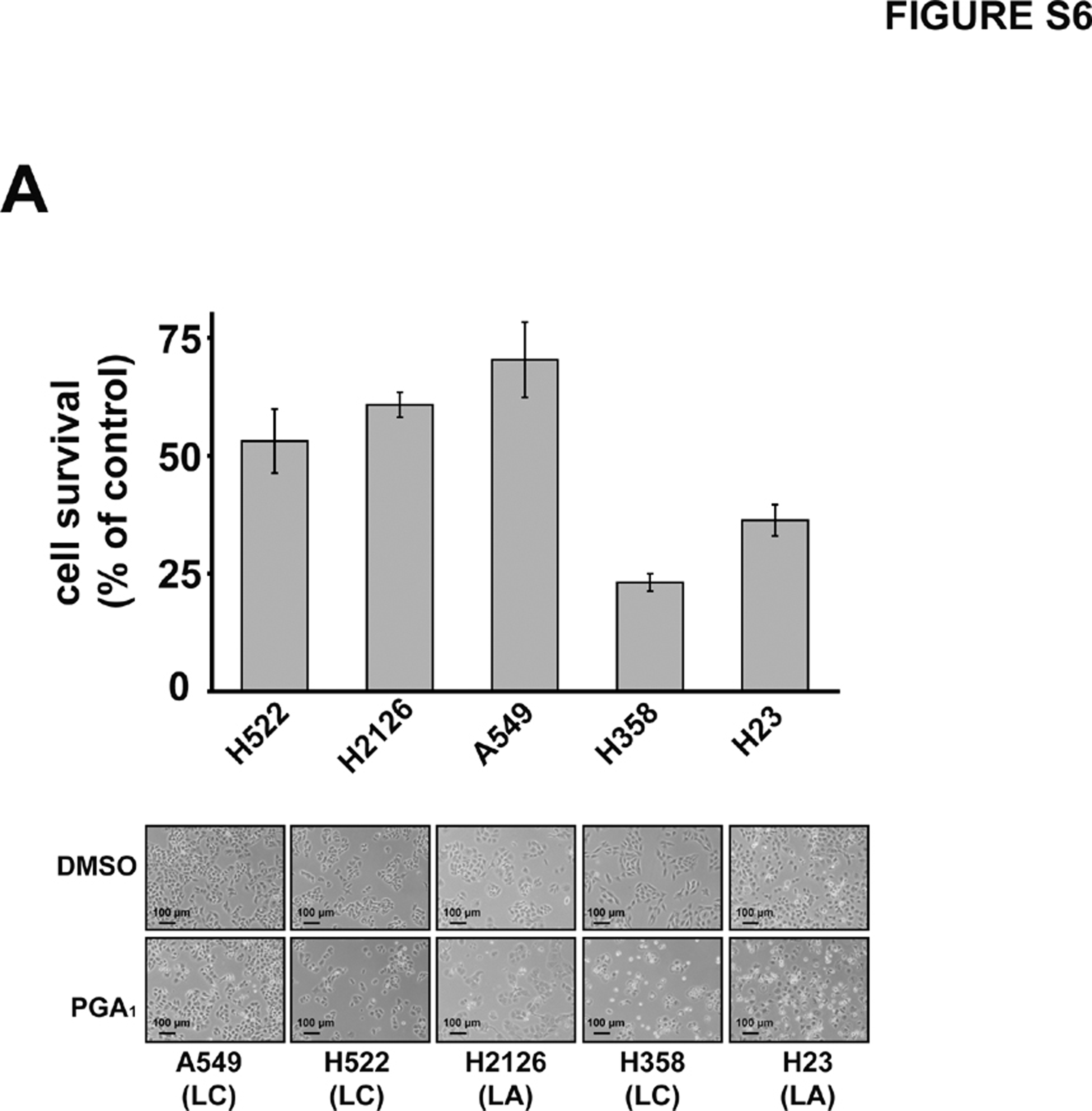

Supplement: Supplementary Figure S5 [file cddis2016219x5.tif]

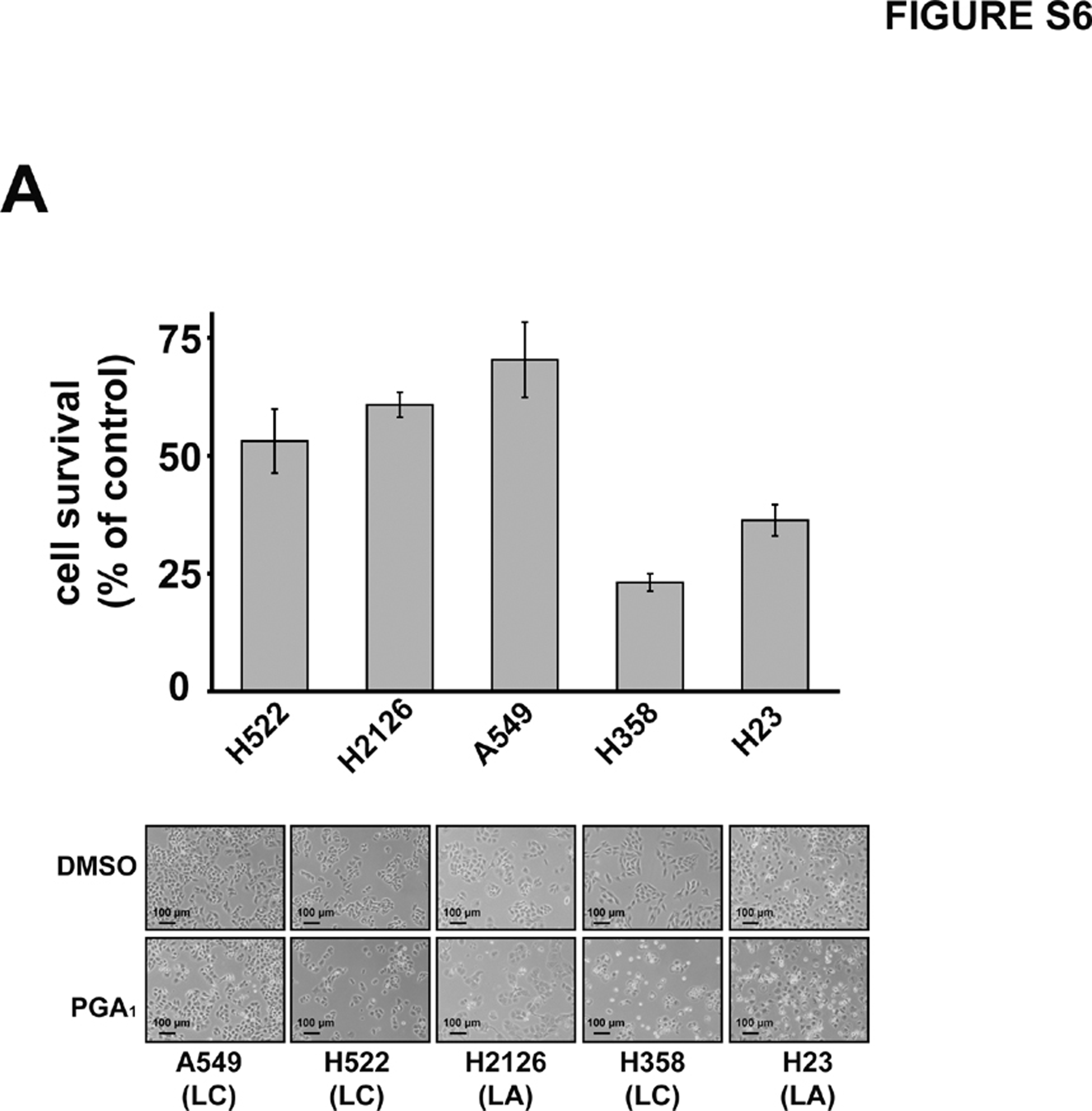

Supplement: Supplementary Figure S6 [file cddis2016219x6.tif]

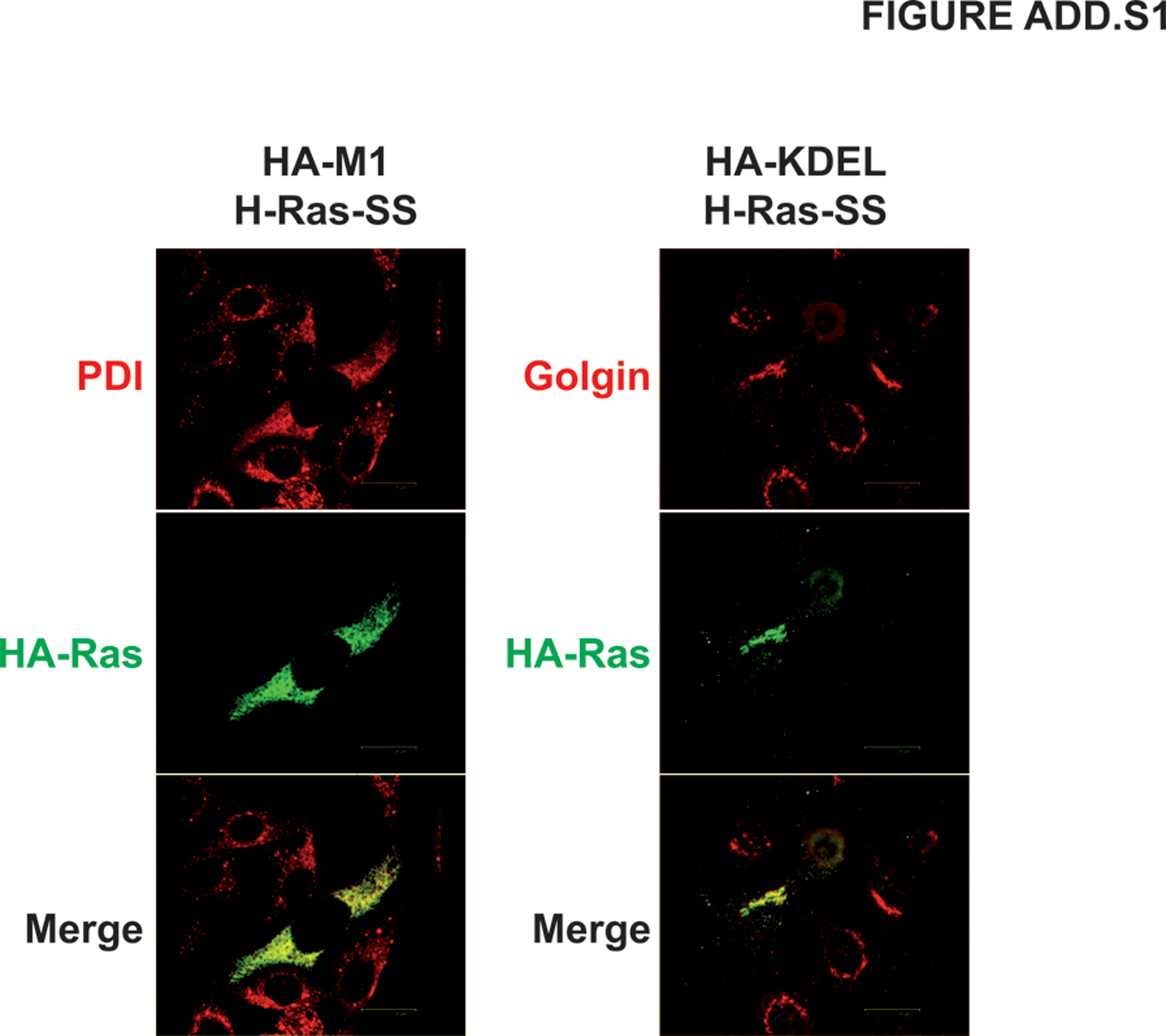

Supplement: Supplementary Figure Add S1 [file cddis2016219x8.tif]

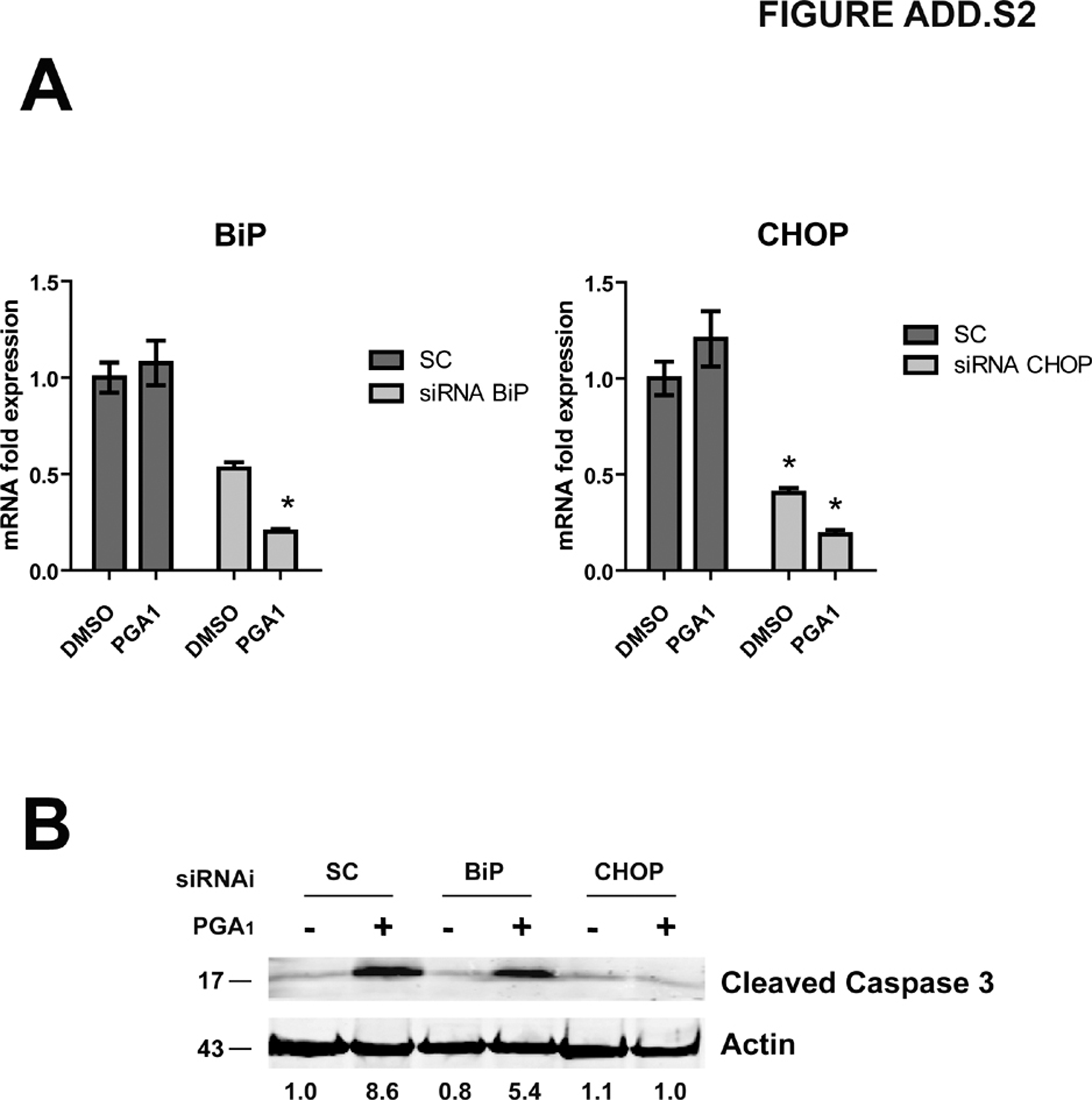

Supplement: Supplementary Figure Add S2 [file cddis2016219x9.tif]
