# Supplementary material for: PGA1-induced apoptosis involves specific activation of H-Ras and N-Ras in cellular endomembranes
Source: Cell Death Dis. 2016 Jul 28;7(7):e2311–. doi: 10.1038/cddis.2016.219 (PMC4973357; doi:10.1038/cddis.2016.219)
Supplement: Supplementary Figure Legends [file cddis2016219x7.doc]

**MS # CDDIS-15-1008-T**

**SUPPLEMENTARY FIGURES**

**Figure S1**. **A**) Schematic representation of the protocol followed in most studies. PGA_1_ was added 24 h after serum deprivation, which corresponds to the G0/G1 phase. In cell proliferation assays, the medium was removed every 24 h, and fresh medium plus PGA_1_ was added. **B**) Analysis of sub-G1 (hypodiploid) DNA content in MEFs 24 h after addition of 30 µM PGA_1_ or DMSO; results are expressed as mean ± SD (n=5). **C**) *Upper panel*, a representative picture of MEFs cells after DAPI staining. Nuclear morphology is shown 24 h after treatment with DMSO or with 30 µM PGA_1_. Arrows highlight the apoptotic nuclei. *Lower panel*, the percentage of apoptotic nuclei was determined by DAPI staining 24 h after addition of DMSO or 30 µM PGA_1_. The incidence of apoptosis was analyzed by counting 500 cells followed by the determination of apoptotic cells in each preparation; results are expressed as mean ± SD of five independent experiments. **D**) Low-molecular-weight DNA was isolated and fractionated on agarose gels 24 h after treatment with 30 µM PGA_1_ or DMSO (see “Methods”). ***P≤0.001 compared with non-stimulated (DMSO) cells.

**Figure S2**. **A**) Analysis of Ras isoform protein levels in MEFs. Starved cultures of MEFs were treated with 30 µM PGA_1_ or DMSO and harvested 3 h after treatment. Cell lysates were analyzed using Western blotting for H-Ras, N-Ras, and K-Ras. Detection of β-actin (actin) was used as a loading control. Similar results were observed in three independent experiments. **B**) H-Ras^-/-^/N-Ras^-/-^ MEFs transiently transfected with pCEFL-KZ-AU5 (Vector), pCEFL-KZ-AU5-H-Ras-wt (H-Ras-wt), pCEFL-KZ-AU5- N-Ras-wt (N-Ras-wt), pCEFL-KZ-AU5-H-RasC184S (H-RasC184S) or pCEFL-KZ-AU5-H-Ras-C118S (H-RasC118S) were serum-starved for 24 h and then incubated with vehicle (-) or 30 µM PGA_1_ (+) and cell morphology was recorded by phase-contrast micrograph 24 h after treatment. All experiments were carried out 3 times with similar results.

**Figure S3. PGA_1_ induces Ras-ERK activation and apoptosis in CH7C17 cells.** **A**) H-Ras^-/-^/N-Ras^-/-^ MEFs transiently transfected with pCEFL-KZ-AU5, pCEFL-KZ-AU5- H-Ras-wt (H-Ras-wt) or pCEFL-KZ-HA-M1-H-RasSS, were serum-starved for 24 h and then incubated with vehicle (-) or 30 µM PGA_1_ (+) and cell morphology was recorded by phase-contrast micrograph 24 h after treatment. All experiments were carried out 3 times with similar results. **B**) Serum-starved CH7C17 cells were stimulated with DMSO, anti-CD3 (5 µg/ml), or PGA_1_ (30 µM). N-Ras-GTP levels were assessed by pull-down assays. Levels of N-Ras activation, which were determined by densitometry are provided at the bottom of the Western blot (SD < 10% average in each case, n=3). **C**) Annexin V/FITC staining analysis of CH7C17 cells stimulated with DMSO or PGA_1_ (30 µM). Results (mean ± SD, n=3) are expressed as a fold increase over the percentage of annexin V–positive cells after incubation with DMSO. **P≤0.01 compared with non-stimulated (DMSO) cells. **D**) Lysates prepared from CH7C17 cells previously stimulated with DMSO, anti-CD3 (5 µg/ml), or PGA_1_ (30 µM for indicated times) underwent Western blotting for ERK and p-ERK. Levels of ERK1/2 activation, which were determined by densitometry, are provided at the bottom of the Western blot (SD < 10% average in each case, n=3).

**Figure S4. Analysis of PGA_1_-elicited ERK activation.** **A**) Time-course analysis for PGA_1_. Wild type MEFs were stimulated with 30 µM PGA_1_ or DMSO and harvested at the indicated times. **B**) Dose-response analysis for PGA_1_. Wild-type MEFs were stimulated with EGF (100 ng/ml) or different concentrations of PGA_1_ and harvested 15 min after treatment. **C**) Serum starved cultures of MEFs treated with 30 µM PGA_1_ or DMSO in the presence of the MEK1/2 inhibitor U0126 (5 µM) added 50 min before PGA_1_ treatment and left in the medium until processing. Values indicate the fold induction in pERK levels normalized to total ERK levels and DMSO, as determined by densitometry (SD < 10% average in each case; n=4). **D**) Apoptosis induced by hyperactive forms of H-Ras. WT MEFs transfected with indicated plasmids were serum starved 24 h after transfection; harvested 24 h later and annexin V/FITC staining analysis was performed. Results (n=4; mean ± S.D.) are expressed as percentage of annexin V positive cells.

**Figure S5. Cytotoxicity of PGA_1_ in cancer cells.** **A**) Cells (2.5 x 10^5^) were seeded in 60-mm dishes in triplicate. After serum starvation during 16 h, cells were treated with 30 µM PGA_1_ or DMSO for 24 h and cells were trypsinized and counted with a hemocytometer. Data are expressed as mean ± SD (n=3). Serum-starved wt or H-Ras^-/-^/N-Ras^-/-^ MEFs **B**) or H358 and A549 cells **C**) were stimulated with 30 µM PGA_1_ or DMSO and cell lysates were obtained 3 h after treatment and were analyzed using Western blot for detection of BiP, CHOP and actin. Similar results were observed in three independent experiments.

**Figure S6. PGA_1_ triggers apoptosis in cancer cells.** H522, H2126, A549, H358 and H23 cells (2.5 x 10^5^) were seeded in 60-mm dishes in triplicate. After serum starvation during 16 h, cells were treated with PGA_1_ 30 µM or DMSO for 24 h and upper cells were trypsinized and counted with a hemocytometer. Abbreviations: LC, Lung Carcinoma; LA, Lung Adenocarcinoma. Data are expressed as mean ± SD (n=3). Lower cell morphology, as determined by phase-contrast microscopy 24 h after PGA_1_ treatment.
