# Supplementary material for: PGA1-induced apoptosis involves specific activation of H-Ras and N-Ras in cellular endomembranes
Source: Cell Death Dis. 2016 Jul 28;7(7):e2311–. doi: 10.1038/cddis.2016.219 (PMC4973357; doi:10.1038/cddis.2016.219)
Supplement: Supplementary Additional Figure Legends [file cddis2016219x10.doc]

**MS # CDDIS-15-1008-T**

**LEGENDS OF ADDITIONAL FIGURES ONLY FOR THE REVIEWERS**

**Figure Add S1. Subcellular localization of targeted Ras proteins.** Representative confocal micrographs of HeLa cells transiently transfected with the indicated plasmids (pCEFL-KZ-HA-M1-H-RasSS or pCEFL-KZ-HA-KDEL-H-Ras-SS). Samples were immunostained with anti-HA (green), and PDI for ER (red), or Golgin-97 for Golgi complex (red). Bars: 20 µm.

**Figure Add S2. Effects originated by the siRNA of BiP and CHOP on the cellular responses elicited by PGA1.** Wild-type MEFs were transfected twice with a mix of 3 different siRNA duplexes of CHOP, BiP, or scrambled (SC) 48 h before serum starvation, and them treated with PGA_1_ or DMSO and harvested 3 h after treatment. **A**) Quantitative RT-PCR analysis of BiP and CHOP mRNA levels. The data are expressed as the mean ± SD (n=3). *P≤0.05 vs. SC (DMSO). **B**) Cell lysates were obtained after treatment and were analyzed using Western blot for detection of cleaved caspase 3 and actin. Levels of cleaved caspase 3 are provided at the bottom (SD < 10% average in each case, n=3).
