# Supplementary material for: PGA1-induced apoptosis involves specific activation of H-Ras and N-Ras in cellular endomembranes
Source: Cell Death Dis. 2016 Jul 28;7(7):e2311–. doi: 10.1038/cddis.2016.219 (PMC4973357; doi:10.1038/cddis.2016.219)
Supplement: Supplementary Information [file cddis2016219x11.doc]

**MS # CDDIS-15-1008-T**

**ADDITIONAL METHODS OF THE FIGURES ONLY FOR THE REVIEWERS**

***siRNA Cell transfection studies.*** Transient siRNA transfection was performed in MEFs using Lipofectamine® RNAiMAX reagent (Invitrogen cat. 13778-075) according to the manufacturer's instructions. Briefly 350.000 cells were transfected with a mix of 3 different gene-specific 27 mer siRNA duplexes (10nM each) of CHOP; SR 403887, BiP; SR 4187499 or scrambled; SR 30004 (Origene Technologies, Inc. Rockville, MD). 24 h after a second transfection was performed. Cells were stimulated with DMSO or PGA1 48 h after and collected for western blot analysis.

***Quantitative RT-PCR***

Total RNA from transfected cells with Scramble, BiP or CHOP was extracted according to the manufacturer’s protocol, Macherey-Nagel Kit (Ref. 740995). Total RNA (2μg) from each sample was reversed using Ready-To-Go-You-prime-First-Strand-Beads (GE Healthcare Ref 27-9264-01). The qRT-PCR was performed with the Go Taq qPCR master mix and the Applied Biosystems 7500 Fast Thermocycler. Primer sequences: Mm Bip Forward: ATCTTTGGTTGCTTGTCGCT; Mm Bip Reverse: ATGAAGGAGACTGCTGAGGC; Mm CHOP Forward: GACCAGGTTCTGCTTTCAGG; Mm CHOP Reverse: CAGCGACAGAGCCAGAATAA; Mm GAPDH Forward: CGTCCCGTAGACAAAATGGT; Mm GAPDH Reverse: TTGATGGCAACAATCTCCAC.

***Confocal immunoflurescence***

Cells were plated on coverslides placed on 35-mm dishes transfected with pCEFL-KZ-HA-M1-H-RasSS or pCEFL- KZ-HA-KDEL-H-Ras-SS and after 24 h cells were washed twice with PBS, fixed for 10 min with methanol, washed three times for 5 min with PBS, and permeabilized for 15 min with 0.25% Triton X-100 in PBS, followed by a 10-min incubation in 100 mM glycine in PBS. After blocking for 30 min with 3% fetal bovine serum in PBS, a primary antibody was added (1 h), followed by washing with 0.1% Tween-20 in PBS, and incubation with a secondary-conjugated. Golgin-97 (CDF4) used at 1 µg/ml Cat # A21270 Invitrogen, Thermo Fisher Scientific (Rockford, IL). PDI antibody (H-160) used at 1.50 dilution (Santa Cruz Biotechnology Inc, Santa Cruz, California, USA; sc-20132), Texas red conjugate used at 1 µg/ml Cat # T-6391 Invitrogen, Thermo Fisher Scientific (Rockford, IL), HA.11 clone 16B12 FITC labelled used at 1.1000 dilution cat# FITC-101L(Berkeley Antibody Company, Berkeley, California, USA). Coverslides were washed three times with PBS, mounted with Vectashield, and visualized with a Confocal Spectral microscope, Leica TCS-SP5. A Lens 63× /1.4 oil was used for all recordings.
